# Parenchymatous Aspiration—A New Method of Diagnosis

**Published:** 1893-12

**Authors:** Albert Abrams

**Affiliations:** San Francisco, Cal.


### PARENCHYMATOUS ASPIRATION—A NEW METHOD OF DIAGNOSIS.

BY ALBERT ABRAMS, M. D., SAN FRANCISCO, CAL.

During the past year I have occasionally occupied myself in investigating a method of diagnosis which I have specified as *parenchymatous aspiration.* This report is preliminary in character, as the results thus far attained with this method of diagnosis are incomplete. The method consists essentially of withdrawing by means of a hypodermic syringe, certain products from pathological formations in solid tissues, and the examination of these products by the microscope. The syringe which I use has a large barrel with a capacity of ten grains, the object of the large barrel being to secure better suction. The packing is of asbestos and the needle is made of platinum. The syringe is constructed on thoroughly antiseptic principles. After use, the syringe is washed with absolute alcohol and the platinum needle heated in the flame of a lamp. With these precautions the danger of septic contamination is reduced to a minimum. In my experiments, in order to secure greater suction, an aspirating apparatus was also used, which for special cases is of undoubted value. The only method to my knowledge which bears any resemblance to parenchymatous aspiration is that known as *akidopeirastik,* which was first brought to the attention of the profession by Middeldorpf, of Breslau, in 1856. Middeldorpf used grooved needles for tissues with fluid contents and harpoons for solid tissue. My method removes products by aspiration only, and unlike the harpoon, no destruction of tissue results other than that produced by a needle ; an important desideratum in visceral diagnosis. Parenchymatous aspiration was first suggested in the following case, which is the analogue of other cases frequently met with by physicians. The case was that of a young man, with pronounced evidence of apical consolidation, whose sputa after repeated examinations with approved methods, failed to show the presence of the bacillus tuberculosis. It was only after the introduction of the hypodermic needle and the removal from the consolidated lung of certain products in which the bacillus tuberculosis, was positively made. Before further experimentation on the living subject, aspiration was made with many fresh pathological specimens with results justifying the clinical application of the method for purposes of diagnosis. With the object of demonstrating the innocuousness of punctures made with the needle in the viscera, rabbits were punctured by my student and myself, and all we noted were striated hemorrhages limited to the course pursued by the needle in the tissues. Necroscopical examination of the lungs of a patient who died of pneumonia, and in whom punctures were made during life, showed identical changes. Punctures made in the lung of the frog and observed under the microscope, demonstrated after removal of the needle, immediate and complete closure of the punctured points. The closure of the punctured points was primarily effected by the inherent contractility of the tissue, and secondarily, by extravasation of the blood.

While the puncture of a physiological lung is practically without danger, it might be urged on theoretical grounds, that the same procedure adopted with a pathological lung would be attended with less favorabie results. The only evidence which I can adduce in refutation of this argument is, that I have punctured the pathological lung more than fifty times for diagnostic, but mainly for experimental purposes, and the only ill effect observed were syncope in one case, hæmoptysis lasting a day in another case and circumscribed pains limited to the punctured points of short duration in two other cases. Parenchymatous aspiration was made with lungs the seat of tuberculosis, pneumonia and syphilis. In undoubted cases of tuberculosis of the lungs and bronchial lymphatic glands, the bacilli of tuberculosis could only be demonstrated in the aspiration products in about 40 per cent. of the individuals punctured. My failure to demonstrate the bacilli in all cases was largely attributable to errors in "technique," which were partially removed after further trials. The following errors were noted: 1. Inability to aspirate pathological products. 2. Indistinctness and granular appearance of the specimens examined. 3. The employment of short needles of too small calibre. The first error I sought to eliminate by separating the needle from the barrel of my syringe, filling the latter with one or two drops of distilled water and discharging the fluid through the needle in which were presumably lodged the aspirated products. If this procedure did not suffice in securing sufficient material for microscopical examination, then the following method was adopted: The syringe having been partially filled with an indifferent solution of salt was injected into the punctured tissues, the needle remaining *in situ*, after waiting until the fluid had presumably taken up sufficient pathological products, it was then withdrawn. It was found that aspiration was assisted by the employment of a syringe with a large barrel. The second error was in a measure obviated by mixing the aspirated products with a drop of albumin on an object glass and staining in the usual way. The third error was eliminated by using a long needle with a commensurately large calibre. The number of badilli found in the specimens examined was, as a rule, small, although in a few instances veritable colonies of the bacilli could be seen. Usually no relation existed between the number of bacilli in the sputa and aspirated products. With the aspirated products in which no bacilli were found, I hoped by culturing these products to prove their presence, but my results in this direction were negative. Only two cases of croupous pneumonia were punctured, in both of which pneumococci were found. Punctures made in two cases of apparent tuberculosis of joints failed to show the bacilli of tuberculosis; while in a case of tuberculosis of the cervical lymph glands, the bacilli of tuberculosis were found, making the provisional diagnosis certain. My limited observations with cases of surgical tuberculosis was occasioned by the little material at my disposal. Through the courtesy of the health officer of San Francisco, Dr. Keeney, and the kindness of the city physician, Dr. Yemans, material at the leper hospital was placed at my disposal. By aspiration the *bacillus lepræ* was invariably found in the leprous nodules, infiltrations and neighboring lymphatic glands. In order to disclaim priority with this method of diagnosis in leprosy, I must here refer to the investigations of Dr. Manson (*The Lancet*, August 23, 1884). This observer recommended the following method of demonstrating the presence of *bacillus lepræ* in infilrated leprous patches by compressing the infiltrated patch in the jaws of an ordinary thin-bladed pile clamp. This has the effect of drawing out the blood from the included tissues, and then the patch is pricked with a needle and the exuded fluid is stained for baccilli.

This method I have modified in examining leprous patches on the extremities by employing a Martin's rubber bandage for rendering the tissues bloodless. The method of Manson is inapplicable in leprosy of the lymph glands and viscera. Enlargement of the inguinal lymphatic glands of syphilitic origin were punctured in ten different cases, and the products stained according to the method of Giaconi, showing supposed syphilis bacilli in two cases. The difficulty of finding the bacilli in so few cases, tallies in the main with their frequency in hard chancres. I punctured the spleen for the *plasmodium malariæ* in malaria, and for the *bacillus typhosus* in typhoid fever; but, as my observations in these cases have been very limited, I hesitate to note results. Through the courtesy of Dr. Ellinwood, I was enabled to puncture carcinomata with gratifying results in the diagnosis of these neoplasms. With specimens of amyloid degeneration and sarcoma, the products obtained by aspiration were sufficient in few instances to establish a diagnosis. With the former material, the micro-chemical reaction of the amyloid substance, could be obtained. The following hypothetical and proven conclusions may be formulated:

1. Parenchymatous aspiration is, when conducted with antiseptic precautions in all new formations not necessitating visceral puncture, a harmless procedure.

2. Parenchymatous aspirations, conducted with normal viscera, is rarely attended with danger, owing to the elasticity of physiological tissues.

3. Hemorrhage may possibly follow aspiration in disease of the viscera, owing to diminished tissue elasticity and pathological change in the blood vessels.

4. The necessity for a correct diagnosis justifies, in properly selected cases, the slight risks attending parenchymatous aspiration.

5. Parenchymatous aspiration is of value in the diagnosis of plumonary tuberculosis with lung consolidation, in which the sputa fail to reveal the presence of the bacillus tuberculosis, as in obstruction of the bronchus leading to the consolidated area, or when the sputa are swallowed as occurs in children, or when the sputa are expectorated with difficulty as in senile individuals.

6. In pulmonary tuberculosis, when consolidation is present, but no sputum, parenchymatous aspiration may furnish the earliest evidence of the disease.

7. Parenchymatous aspiration may be of value in the differential diagnosis of pulmonary consolidations when an examination of the sputum proves negative.

8. In surgical tuberculosis of the lymph glands, bones, skin, testicles and other structures, parenchymatous aspiration is of undoubted importance.

9. Parenchymatous aspirations may be of value in the diagnosis of tumors either superficial or deep-seated.

10. In amyloid degeneration of the viscera, parenchymatous aspiration may furnish the only evidence of the affection.

11. In malaria, typhoid fever and other infectious diseases, puncture of the spleen may prove of inestimable value in diagnosis.

12. Leprous regions of the external parts or viscera, may be correctly diagnosed by the aid of parenchymatous aspiration.

13. The lesions of syphilis may be determined by means of this method.

14. Commensurate with our advanced knowledge in the domain of bacteriology and the employment of methods for the more ready recognition of pathogenic microbes, parenchymatous aspiration, as a diagnostic measure, will enhance in value.

